# Automated Bone Age Assessment: A New Three-Stage Assessment Method from Coarse to Fine

**DOI:** 10.3390/healthcare10112170

**Published:** 2022-10-30

**Authors:** Xinzheng Xu, Huihui Xu, Zhongnian Li

**Affiliations:** School of Computer Science and Technology, China University of Mining and Technology, Xuzhou 221116, China

**Keywords:** bone age assessment, deep learning, object detection, convolutional neural network, fine-grained image classification

## Abstract

Bone age assessment (BAA) based on X-ray imaging of the left hand and wrist can accurately reflect the degree of the body’s physiological development and physical condition. However, the traditional manual evaluation method relies too much on inefficient specialist labor. In this paper, to propose automatic BAA, we introduce a hierarchical convolutional neural network to detect the regions of interest (ROI) and classify the bone grade. Firstly, we establish a dataset of children’s BAA containing 2518 left hand X-rays. Then, we use the fine-grained classification to obtain the grade of the region of interest via object detection. Specifically, fine-grained classifiers are based on context-aware attention pooling (CAP). Finally, we perform the model assessment of bone age using the third version of the Tanner–Whitehouse (TW3) methodology. The end-to-end BAA system provides bone age values, the detection results of 13 ROIs, and the bone maturity of the ROIs, which are convenient for doctors to obtain information for operation. Experimental results on the public dataset and clinical dataset show that the performance of the proposed method is competitive. The accuracy of bone grading is 86.93%, and the mean absolute error (MAE) of bone age is 7.68 months on the clinical dataset. On public dataset, the MAE is 6.53 months. The proposed method achieves good performance in bone age assessment and is superior to existing fine-grained image classification methods.

## 1. Introduction

**Figure 1:**
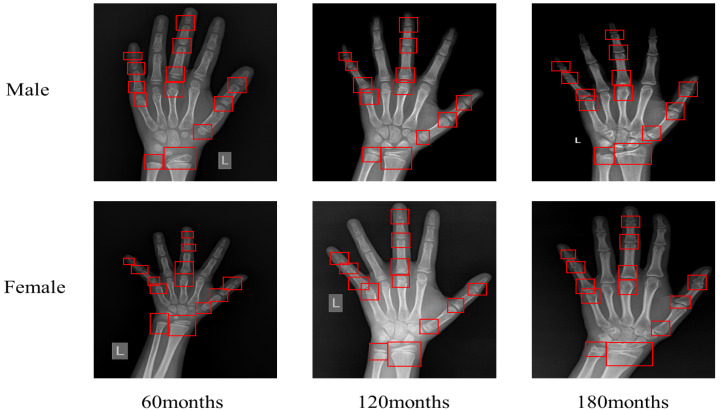
Left hand X-ray images of male and female children at different ages. The red boxes are the ROIs.

Human growth and development age can be divided into calendar age and biological age. Biological age is generally judged by bone age medically. Different bones of the human body show continuity and stages in the development process. Figure 1 shows typical X-ray pictures of hands and wrists at different ages; it can be seen in the figure that there are obvious differences in bone morphology at different ages. Bone age [1] is determined by observing the developmental status of the left hand bone, such as its shape, size, location, and degree of closure. Bone age assessment (BAA) was first applied to medical research to help understand and promote children’s growth retardation, prevent precocious puberty, predict children’s adult height, and assist in diagnosing endocrine diseases. Subsequently, the application scenarios were widely expanded, such as determining the actual age of athletes and forensic science as a criterion for no conviction [2].

The original BAA was based on a left hand X-ray, and bone age was assessed by physicians by manual reading by the Greenich and Pyle (GP) [3] method or the Tanner–Whitehouse (TW) method. The GP atlas method compares the image to the reference atlas and selects the most similar profile for its estimated bone age. The TW method uses cumulative scores for the developmental morphology of different bones to assess bone age. The latest version of the TW3 method is more objective than the GP mapping method, so it is considered to have higher accuracy and reproducibility than the GP mapping method [4,5]. However, the BBA method by orthopedic specialists is not only cumbersome and time-consuming, but also has differences between observers. Therefore, it is of great significance to research and develop an automatic accurate, stable, simple, inexpensive, and rapid method for end-to-end automated BBA.

To overcome the above difficulties, we propose a novel BAA framework to achieve end-to-end BAA. The three-stage hierarchical assessment method cascades the convolutional neural network of object detection and fine-grained classification. Firstly, to improve the accuracy of the assessment, the YOLOv5 object detection model is used to extract 13 ROIs, which is the R (radial, ulnar, metacarpal, and phalangeal bones) series in the TW3 method. Secondly, because the difference between different bone ages of the same bone is small, the use of a simple classifier for classification has a low accuracy rate, and the fine-grained classification method solves this problem very well. Finally, estimates are made using the recognized TW3 method to enable the end-to-end automated BAA system. Experiments on our clinical datasets verified the superiority of the proposed three-stage hierarchical BAA method.

The contributions of the proposed methods can be summarized as follows:

1. We design a novel framework and propose a three-stage hierarchical assessment method based on object detection and fine-grained classification for BAA.

2. We use fine-grained image classification based on context perception to grade 13 bones, resulting in higher accuracy in bone age classification.

3. Experiments on clinical datasets prove that the proposed three-stage hierarchical model based on object detection and fine-grained classification has excellent evaluation performance compared with the most-advanced methods.

## 2. Related Works

In recent years, deep learning has gradually been applied to the field of medical imaging due to its powerful properties and, in some cases, has reached or even exceeded the expert level. Therefore, researchers have begun to apply convolutional neural networks to BAA to improve the efficiency and accuracy of BAA.

### 2.1. BAA via Deep Learning

The existing end-to-end BAA detection methods in China generally only output an estimated bone age value, which lacks the interpretability of the model. Spampinato et al. [6] proposed a lightweight convolutional neural network model BoNet consisting of five convolutional layers, one pooling layer, and one fully connected layer that could be assessed for bone age across races, age ranges, and sexes, yielding an average difference of 9.48 months. Lee et al. [7] normalized the images using pre-processing, followed by a BAA using fine-tuned GooleNet, and generated structured radiology reports based on the doctor’s final decision, with an accuracy rate of 61.40% for males and 57.32% for females, respectively. In another work, Son et al. [8] used faster R-CNN to segment 13 ROIs from left hand X-ray images and then trained the VGG network using ROIs extracted from 3344 images, which differed from the experts by an average of 5.52 months.

Alexander et al. [9] won first place in the Pediatric Bone Age Machine Learning Challenge organized by the North American Radiological Society (RSNA) for the Inception V3 [10] architecture for pixel information in tandem with gender information, with an additional dense layer after concatenation to enable the network to learn the relationship between pixel and gender information, obtaining a mean absolute error (MAE) of 4.27–4.5 months on the RSNA dataset. Yang et al. [11] proposed to perform fine-grained precision assessment by integrating prior knowledge and a pyramid network of recursive features, and the MAE of 1–18 years old was 7.32 months. Liu et al. [12] constructed a two-stage automated assessment method that cascaded coarse-to-fine hands and an integrated convolutional neural network cascade based on stacking, and 5.42/6.58 months of MAE for both males and females was achieved on the RSNA dataset. The above study shows that the automatic BAA method based on deep learning can achieve considerable accuracy. Radiologists focus not only on bone age values, but also on detailed information about bone development. However, the above studies only provide bone age values, which cannot meet the needs of doctors.

### 2.2. Object Detection

Convolutional neural networks (CNNs) are the simplest and most widely used deep learning algorithms for object detection, which are mainly divided into one-stage and two-stage methods. One-stage algorithms extracts feature directly from the network to predict object classification and location. Redmon et al. [13] proposed YOLO’s first-stage deep learning detection algorithm in 2016, currently available in five versions, which are optimal in terms of speed and results. The SSD algorithm, proposed by Liu et al. [14], raises the speeds by eliminating the need for a regional recommendation network, while improving detection accuracy by applying multi-scale features and default boxes.

The two-stage first generates a region proposal (a pre-selection box that may contain the object to be detected) and then classifies the samples through a convolutional neural network. For example, Uijlings et al. [15] proposed a region-based convolutional neural network (R-CNN) in 2013, which is mainly divided into four steps: candidate region generation, feature extraction, category judgment, and position refinement, which is the pioneering work of introducing the CNN into object detection. This was followed by Fast R-CNN [16] and Faster R-CNN [17], which became the leaders in object detection. The two methods can be combined: Zhang et al. [18] proposed a two-way parallel feature pyramid network to effectively capture multi-scale spatial information from all levels of the feature pyramid network (FPN), achieving better performance than other FCNs.

Wang et al. [19] proposed end-to-end object detection based on a fully convolutional network, which introduces predictive perception one-to-one label assignment for classification to achieve end-to-end detection. In addition, a simple three-dimensional maximum filter (3DMF) was proposed to improve the observance of local regional convolution by using multi-scale features, and it showed good performance on coco and other datasets. Now, target detection technology has become very mature and is gradually being used in the field of medical image processing.

### 2.3. Fine-Grained Image Classification

The problem of fine-grained image classification is to identify sub-classes under large categories, and the granularity of the categories to which fine-grained images belong is finer, while the differences within classes are large; however, the differences between classes are small, so classification is more challenging [20]. At present, fine-grained classification methods are mainly divided into two categories: strongly supervised classification methods and weakly supervised classification methods.

Strong supervised fine-grained classification requires global and local information for fine-grained classification, that is not only category labels, but also local annotation information such as the bounding box and part annotation. Zhang et al. [21] used the R-CNN algorithm to detect the object level (such as all kinds of birds) and its local areas (head, body, and other parts) of fine-grained images. Their proposed model, partly based on the R-CNN, uses both object-level features and local features in the classification process, so the classification accuracy is relatively ideal. Wei et al. [22] proposed the Mask CNN model, which is divided into two modules. The first is part localization, and the second is feature learning of global and local image blocks. The difference from the previous method is that this method proposes that the FCN learn a part-based segmentation model by using the fully connected network. This method achieved the highest classification accuracy of 87.3% for fine-grained image classification at that time.

The fine-grained classification model with weak supervision information uses the attention mechanism, clustering, and other means to enable the model to automatically find distinctive regions and only uses classification tags to complete training. Lin et al. [23] proposed a recognition structure called “bilinear CNN”, which uses bilinear model fusion features for classification. Yang et al. [24] proposed that the navigator–teacher–scrutinizer network (NTS-Net) be a weakly supervised network that integrates the overall features with the region with the largest amount of information extracted from the original image without using only category labels. Liu et al. [24] proposed a mixed-attention network (SMA-Net), which mainly uses the attention network to locate discriminant regions and feature extraction, which effectively improves the performance of the network.

## 3. Method

**Figure 2:**
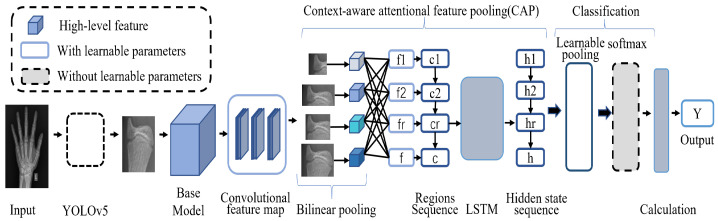
Hierarchical network structure. YOLOv5 is used to predict the bone category, select the corresponding classifier according to the output results, classify the bone grade, obtain the classification results, and evaluate the bone age Y. There are 13 classifiers in total, and one classifier is taken as an example in the figure.

In this section, we introduce our novel BAA framework, a hierarchical network model based on object detection and fine-grained classification; the overall architecture of the entire model is shown in Figure 2, and the ultimate goal is to achieve end-to-end BAA.

### 3.1. Structure of Bone Age Assessment Model

**Figure 3:**
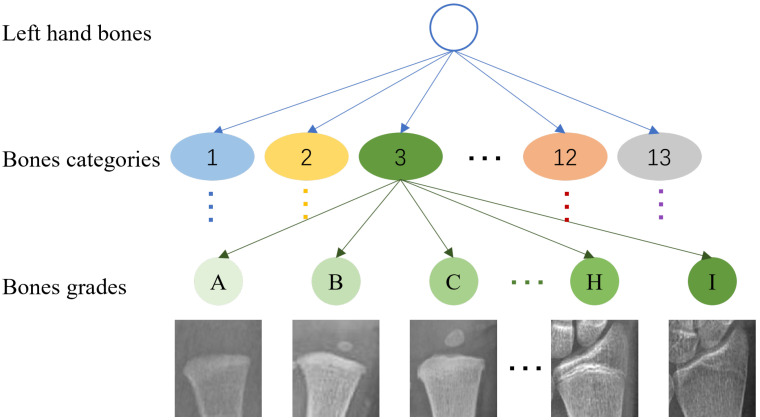
The tree structure of bone categories and grades. The thirteen colors, such as blue, yellow, green, orange, and gray, correspond to the thirteen categories, respectively (ulna, radius, the 1st proximal and distal phalanges, the 3rd proximal, middle, and distal phalanges, the 5th proximal, middle, and distal phalanges, and the 1st, 3rd, and 5th metacarpals). Different shades of each color represent different grades (A, B, C, D, E, F, G, H, I (ulna does not have an I-th grade)).

We divided the bone identification and grades into a three-level tree structure (including the root nodes), as shown in Figure 3. The network first takes the left hand X-ray image as the input, selects the YOLOv5 model for recognition, obtains the prediction result of the bone category, and saves the detection results by cropping. Then, according to the corresponding category, the output images of the previous network are transferred to the corresponding classifier for classification through a selector. In the test phase, the prediction result of the input image bone category is obtained through the detection model, and then, the corresponding branch structure is selected by the selector based on the result. Finally, the bone age is estimated according to the TW3 method based on the 13 bone grade results, and the result is output.

### 3.2. Extraction of 13 ROIs

**Figure 4:**
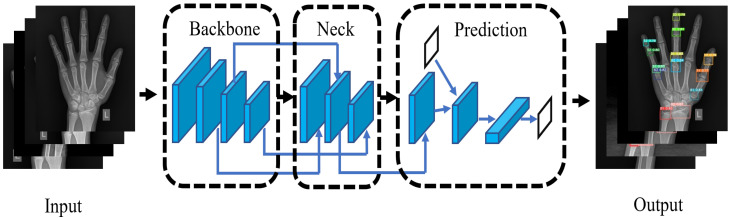
YOLOv5 structure diagram; the black box is the prediction box.

We used YOLOv5 to extract the ROIs from the left hand X-ray image, and the structure diagram of YOLOv5 we used is shown in Figure 4. We input an image and performed mosaic data enhancement, adaptive anchor frame calculation, and adaptive image scaling processing to improve the accuracy and inference speed of small object detection. In the feature extraction stage, the slicing operation and CSP structure are used to strengthen the ability of network feature fusion so that more fine-grained features can be extracted. The network uses the GIOU_Loss [25] function and uses the non-maximum suppression (NMS) method to filter the target frame, outputs 13 bone category detection frames, and saves the results. The algorithm description of GIOU_Loss is shown in Algorithm 1.

**Algorithm 1** Generalized intersection over union [25]
**Input:** 
  Two arbitrary convex shapes: $A,B\subseteq S\in R^{n}$**Output:** 
  GIoU 1 For *A* and *B*, find the smallest enclosing convex object *C*, where $C\subseteq S\in R^{n}$ 2 $IoU=\frac{\left|A\cap B\right|}{\left|A\cup B\right|}$ 3 $GIoU=IoU-\frac{\left|C(A\cup B)\right|}{\left|C\right|}$


### 3.3. Hierarchical Classification of Hand Bones

Since the training of classifiers differs only in the dataset categories, let us take one of the classifiers as an example. Given an image P, input the basic convolutional neural network (Xception) [26], perform feature extraction, and send the output features to the CAP [27] module. Learning the importance of adjusting different areas of features, the module correlates the contextual information of the picture at the pixel level to obtain relevant attention features. To make the classification of the model more accurate, the learnable pooling operation is selected, the feature information is integrated by combining hidden layers with similar responses, the response values of hidden layers are assigned to different class clusters according to the weight of softmax, and all the weights are normalized to output the bone grade classification results.

The loss function is an indicator to measure the performance of the expected results of the prediction model. Denote X as the input space of the training instance and Y={1,2,…,K} as the output label space. Let $D={\left\{\left(x_{i},y_{i}\right)\right\}}_{i=1}^{n}$ be the training instance image, where $x_{i}\subset X$ denotes each training instance image, and $y_{i}\subset Y$ denotes the ground-truth label of $x_{i}$. Classifier f:X→Y from *D*, which aims to predict ground-truth labels from a set of candidate labels. The basic CELoss function is given by:

(1) $$CELoss\left(x,y\right)=-\frac{1}{N}\sum_{i=1}^{N}\sum_{i=1}^{K}y_{i}*log\left(Y_{i}\right)$$

where *N* is the number of samples, *K* is the number of categories, $y_{i}$ is the actual value, and $Y_{i}$ is the predicted value.

## 4. Experiments

### 4.1. Datasets

The datasets used in this study include a public dataset and a clinical dataset. The public dataset came from the 2017 Pediatric Bone Age Challenge organized by the Radiological Society of North America (RSNA), which includes 12.6 k images. Among them, there are 6833 images for males and 5778 images for females.

**Figure 5:**
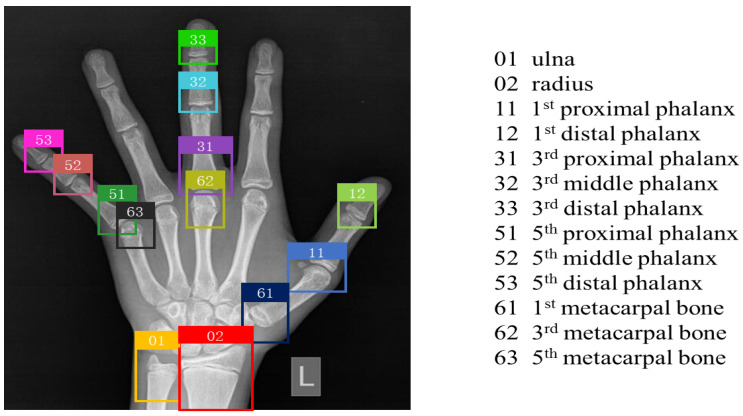
Training set image marked by the doctor. The rectangular box is the callout area, and the differently colored rectangular boxes represent different bone categories.

The clinical dataset came mainly from Xuzhou Central Hospital, which provides 2518 left hand X-ray maps of children under the age of 18. Firstly, we desensitized each image to remove sensitive information from the image content and protect the patients’ privacy. Secondly, a number of professional radiologists marked the pre-treated images with bone categories and bone grades to ensure the accuracy of these data. As shown in Figure 5, the labeling of each picture in the object detection dataset is composed of the category and coordinate information of 13 bones, including the ulna, the radius, the 1st, 3rd, and 5th metacarpal bones, the 1st, 3rd, and 5th proximal phalanges, the 3rd and 5th middle phalanges, and the 1st, 3rd, and 5th distal phalanges. On this basis, we cropped 13 ROIs and labeled them with image-level bone levels, resulting in 32,734 fine-grained classification datasets.

### 4.2. Training Details

**Figure 6:**
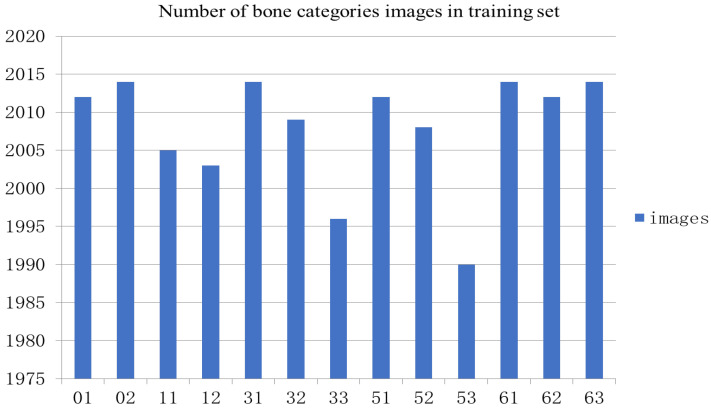
The number of images in the training set. The vertical axis is the number of images, and the horizontal axis is the category of different bones.

The structure diagram of YOLOv5 we used is shown in Figure 4, in the bone class classification experiment. Using 2518 images labeled by the doctor, the training set and the test set were divided into 8:2, and Figure 6 shows the amount of data for each type of dataset. YOLOv5 with 100 epochs was trained using the pre-trained model YOLOYv5s, and the parameters of the model were as follows: the learning rate was 0.01, and the batch size was 16.

The training used an NVIDIA Tesla K80 GPU, 12 GB of graphics memory, and 8 GB of running memory. The CPU model was an Intel(R) Xeon(R) CPU E5-2678 v3 @ 2.50 GHz. The operating systems were Ubuntu 18.04, CUDA 10.0, and cuDNN 7.6. The software versions were Python 3.5, Tensorflow 1.13.1, and Keras 2.2.

The classifier uses fine-grained classification based on context-aware attention pooling, trains 13 classifiers according to different bone categories, uses a dataset of 2518 images, uses the Xcpetion pre-training network, and selects stochastic gradient descent (SGD) [28] to train the model. The training cycle was 100, the learning rate 0.0001, the momentum 0.9, and the batch size 8.

### 4.3. Results

#### 4.3.1. Extraction of 13 ROIs

We selected 13 skeletons that conform to human prior knowledge as recognition positions. By observing the visual results of the training process, it was shown that YOLOv5 is capable of extracting ROIs, which can ensure stable age assessment without additional foreground–background segmentation.

**Figure 7:**
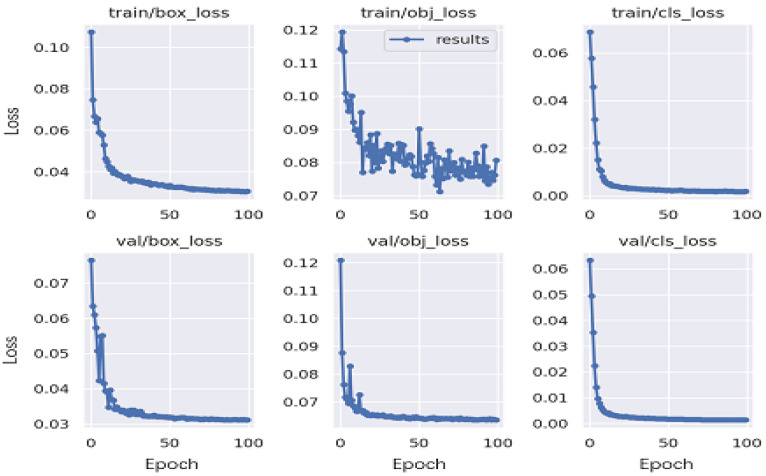
The loss of the model. The first line is the training loss, and the second line is the validation loss. The box_loss is the loss function of the prediction box; the obj_loss is the target detection; the cls_loss is the classification loss.

Figure 7 shows some of the losses during model training. The box_loss is the loss function of the prediction box, and the smaller the value, the more accurate the target box it; Obj_loss is the target detection, and the smaller the target detection, the more accurate the detection is; cls_loss is the classification loss, and the smaller the classification, the more accurate the classification is. Each of the results shown converged appropriately to 0; therefore, it can be concluded that the proposed model acquired as many ROIs as possible.

**Figure 8:**
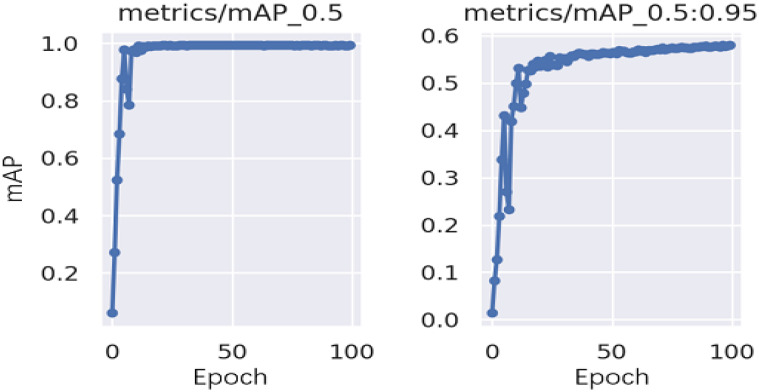
Mean average precision (mAP) graphs for the categories. mAP_0.5 represents the mAP value when the intersection over union (IoU) is 0.5; mAP_0.5:0.95 represents the average mAP at different IoU thresholds (from 0.5 to 0.95 in steps of 0.05) (0.5, 0.55, 0.6, 0.65, 0.7, 0.75, 0.8, 0.85, 0.9, 0.95).

The mean average precision (mAP) results of the training are shown in Figure 8. Precision represents how many of the data predicted as positive samples are true positive samples. The recall is how many positive samples the model retrieves out of the total positive samples. The AP on the PR graph is the area below the PR curve, and the value range is from 0–1; for the accuracy of the model, the higher the recall, then the better the performance of the model, that is, the larger the AP, the better the performance of the model is. As shown in the figure, the mAP for the 13 categories of the model was 0.993, which indicates that the recommended model performs well.

#### 4.3.2. Hierarchical Classification of Hand Bones

To verify the validity of the fine-grained classification model we used, we compared it with other classification models and output bone maturity level results. Table 1 shows the classification accuracy of bone grade classification using different classifiers on our clinical dataset. The results show that, compared with other classification methods, the model we used achieved the highest classification accuracy (the average was 86.93%) in bone grade, which further verifies the superiority of the proposed three-stage automatic pediatric bone age assessment method.

#### 4.3.3. End-to-End Automated BAA

The evaluation index MAE of the bone age assessment method: The MAE formula is defined as follows:

(2) $$MAE=\frac{\sum_{i=1}^{N}\left|R_{i}-P_{i}\right|}{N}$$

where *N* is the number of pictures in the test set, $R_{i}$ represents the true bone age of the *i*-th picture, and $P_{i}$ represents the predicted bone age of the *i*-th picture.

The results of this study were compared with those of the BAA algorithm published in the existing literature and are shown in Table 2. The results showed that the three-stage hierarchical assessment method based on object detection and fine-grained classification proposed by us is superior to the existing methods, and the accuracy is much higher than that of the traditional manual method of assessing bone age.

## 5. Discussion

In this study, our proposed three-stage BAA method achieved relatively satisfactory results on both the clinical dataset and the public dataset RSNA.

In the first stage, YOLOv5 was used to extract 13 ROIs, and the detection accuracy as as high as 98.93%. Most of the studies used Faster R-CNN in the extraction of ROIs, and these studies all had the phenomenon of missing ROI detection [36,37]. In the second stage, CAP was used to perform 13 bone grade staging, and the average accuracy was 86.93%, which was much higher than the 63.15% accuracy achieved by the recursive feature pyramid network proposed by Jia et al. [11]. In the third stage, the TW3 method was used to estimate bone age, which is more accurate than building a regression model of bone age.

At present, most BAA studies based on deep learning only provide an end-to-end bone age assessment result. However, we learned through communication with doctors that radiologists pay more attention to the details of hand bone changes than a simple bone age. Our BAA system not only provides bone age, but also provides the detection results of 13 ROIs, which is convenient for doctors to further observe the information and provide help for the next diagnosis.

In our research, we found that in the dataset samples provided by the hospital, the sample size of 0–5 years old is much lower than that of 6–18 years old, which leads to uneven sample distribution. The imbalance of the sample is likely to affect the results of bone age detection, making the bone age assessment value of 0–6 years old inaccurate. Our next plan is to design a more suitable framework to solve the bone age assessment of imbalanced samples based on the existing basis, so as to make the bone age assessment more accurate.

## 6. Conclusions

Bone age assessment (BAA) based on X-ray imaging of the left hand and wrist can accurately reflect the degree of the body’s physiological development and physical condition. However, the traditional manual evaluation method relies too much on inefficient specialist labor. In this paper, we designed a three-stage hierarchical approach (consisting of object detection, fine-grained classification, and TW3 computation) to implement end-to-end BBA. The framework can obtain detailed information such as bone age values, 13 ROIs, and the maturity level of each bone, which is very good for the needs of doctors. The BAA system has high bone age estimation accuracy and computational speed block and achieved 86.93% classification accuracy and an MAE of bone age of 7.68 months on the clinical dataset, as well as a 6.53-month MAE on the public dataset, to meet clinical needs.

## Figures and Tables

**Table 1:** Accuracy comparisons of different classification models on the clinical dataset.

ROI No.	MLP-Mixer [29]	PMG [30]	WS-DAN [31]	CAP
1	66.59%	79.12%	80.07%	**98.79**%
2	66.25%	75.50%	73.28%	**94.15**%
3	51.30%	71.20%	69.52%	**97.17**%
4	46.73%	62.00%	63.59%	**83.87**%
5	59.69%	66.63%	64.57%	**91.80**%
6	54.50%	49.19%	59.30%	**87.60**%
7	55.32%	64.56%	62.61%	**82.54**%
8	54.10%	63.35%	62.23%	**85.74**%
9	58.52%	63.91%	61.85%	**84.38**%
10	51.05%	60.39%	61.70%	**83.13**%
11	52.96%	61.92%	60.41%	**81.05**%
12	55.12%	60.32%	61.81%	**79.23**%
13	58.07%	61.92%	59.37%	**80.65**%
AVG	56.17%	64.62%	64.64%	**86.93**%

**Table 2:** Comparison with different methods on the RASA dataset.

Evaluation Index	MAE (Months)
Wu et al. [32]	7.38
Steenkiste et al. [33]	6.80
Iglovikov et al. [34]	6.89
BoneXpert [35]	8.16
**YOLOv5+CAP**	**6.53**

## Data Availability

Not applicable.
